# Investigating sex-specific dynamics using uniparental markers: West New Guinea as a case study

**DOI:** 10.1002/ece3.660

**Published:** 2013-07-02

**Authors:** Stefano Mona, Ernest Mordret, Michel Veuille, Mila Tommaseo-Ponzetta

**Affiliations:** 1 Laboratoire Biologie intégrative des populations, Ecole Pratique des Hautes Etudes46 rue de Lille, 75007, Paris, France; 2 CNRS UMR 7205, Muséum National d'Histoire NaturelleRue Buffon, 75005, Paris, France; 3 Department of Biology, University of Bari70100, Bari, Italy

**Keywords:** Gene genealogy, metapopulation, patrilocality, spatial simulation, West New Guinea

## Abstract

Mitochondrial DNA (mtDNA) and Y chromosome (NRY) genetic markers have been often contrasted to investigate sex-specific dynamics. Traditionally, isolation by distance, intrapopulation genetic diversity and population differentiation are estimated from both markers and compared. Two possible sources of bias are often neglected. First, kilometric distances are frequently used as predictor of the connectivity between groups, hiding the role played by environmental features at a microgeographic scale. Second, the comparison of intrapopulation diversity and population differentiation between mtDNA and NRY is hampered by their different mutational mechanisms and rates. Here, we show how to account for these biases by analyzing from a different perspective a published dataset of eight West New Guinea (WNG) populations for which mtDNA control region sequences and seven linked NRY microsatellites had been typed. First, we modeled the connectivity among sampled populations by computing the number of days required to travel between groups. Then, we investigated the differences between the two sexes accounting for the molecular characteristics of the markers examined to obtain estimates on the product of the effective population size and the migration rate among demes (*Nm*). We achieved this goal by studying the shape of the gene genealogy at several sampling levels and using spatial explicit simulations. Both the direction and the rate of migration differ between male and females, with an *Nm* estimated to be >6 times higher in the latter under many evolutionary scenarios. We finally highlight the importance of applying metapopulation models when analyzing the genetic diversity of a species.

We have applied the prediction of the sampling theory in a meta-population and we have corroborated our finding using spatial explicit simulations. Both approaches are fundamentally meant to deal with structured populations: we strongly believe in the importance of tacking structure into account when inferring the demographic history of a species.

## Introduction

Sex-specific demographic patterns are traditionally investigated in humans by using uniparentally inherited markers, namely mitochondrial DNA (mtDNA) for females and Y chromosome (NRY) for males. Reports usually focus mainly on three classes of analysis, whose results are compared between the two markers: (i) isolation by distance (IBD) (Seielstad et al. 48; Wood et al. 67; Delfin et al. 11; Kemp et al. 32); (ii) intrapopulation diversity levels (Oota et al. 42; Kayser et al. 31; Gunnarsdottir et al. 23); (iii) extent of population differentiation (Seielstad et al. 48; Oota et al. 42; Nasidze et al. 40). Most of the evidences presented so far suggest that paternal and maternal histories differ heavily, probably as a consequence of cultural behaviors related to postmarital residential pattern and variance in reproductive success (see Heyer et al. 27 and references therein). However, different conclusions have been drawn depending on the region investigated (Seielstad et al. 48; Oota et al. 42; Fuselli et al. 20; Kayser et al. 31; Nasidze et al. 40; Kemp et al. 32), on the geographic scale of the study (Wilder et al. 63; Wilkins and Marlowe 65) and on the specific locus ascertained on both mtDNA and NRY (Wilder et al. 63; Gunnarsdottir et al. 23).

There are several pitfalls when investigating male and female population genetics patterns. First, despite the advances in next generation sequencing technologies which allow access to an incredibly large amount of data (Metzker 38), comparison of male and female population dynamics still relies on a small and nonrecombining portion of our genome (however, see Balaresque et al. 2; Segurel et al. 47 for exceptions). This limits the possibility of accounting for the large stochastic variance associated with the coalescent process, which can be mitigated through the analysis of as many independent loci as possible (Felsenstein 17). Second, IBD analysis usually relies on simple kilometric distance matrices and does not take explicitly into account environmental features, which can affect connectivity among populations (McRae and Beier 36). Third, population genetic models used often assume equilibrium (i.e., Wright 68 island model), hence ignoring both temporal and spatial heterogeneity. Indeed, human societies have probably changed their lifestyle on multiple occasions. Moreover, the carrying capacity of various habitats has not been constant through time due, for instance, to technological innovations, leading to variation in the effective size of human populations. Fourth, mutation rate influences both the gene diversity of a sample (Hudson 29) and many frequently used measure of population differentiation such as *Fst* and *Rst* (Hedrick 24; Meirmans 37). This must be acknowledged when comparing the results obtained from loci with different mutation rates such as mtDNA and NRY microsatellites.

In this report, we show how to cope with the differences among the markers to be compared and the advantages of taking environmental features into account when modeling genetic diversity. To this end, we analyzed in a new light a published dataset of mtDNA and NRY microsatellites variation in eight West New Guinea (WNG) groups (Tommaseo-Ponzetta et al. 55; Kayser et al. 31). The long human presence in the island as well as the environmental and temporal heterogeneity makes WNG an interesting region to investigate population dynamics and contrast male and female histories. New Guinea was colonized in the last part of the Pleistocene when the island, together with Australia and Tasmania, was part of one paleocontinent called “Sahul”. Although the earliest human presence in the highlands of New Guinea is documented 49,000 years ago (Summerhayes et al. 53), by analogy with Australian prehistoric evidence this occupation may have been even earlier (Roberts et al. 46; Fullagar et al. 19; O'Connell and Allen 41; Thorne et al. 54). The first migrants were hunter–gatherers who probably spread along the coast of the continent, leaving little evidence of their passage. Later, many hunter–gatherer groups in the highland plateaus shifted to agriculture. According to archaeological findings, an independent origin of agriculture in New Guinea can be dated as far back as 10,000–9000 years before present, with more intensive cultivation of various species starting by 7000–6500 years before present in Papua New Guinea (Golson 22; Denham et al. 13; Denham 12).

The analyzed populations cover two regions of WNG with different environmental characteristics: three of the eight groups are farmers, inhabiting the interior highlands, whereas the remaining five are hunter/fisher–gatherers (Tommaseo-Ponzetta et al. 55), inhabiting the lowlands. A reduced NRY diversity compared with mtDNA was already put forward when analyzing these eight groups (Kayser et al. 31), making them suitable for more in-depth comparison. The differences between the two sexes were mostly explained as a by-product of patrilocality and polygyny (Kayser et al. 31) (recently, however, Heyer et al. 27 showed that polygyny alone has a limited influence on male effective population size), but no formal hypothesis testing was carried out to obtain quantitative estimate on their intensities.

Here, we took environmental conditions into account by recording the walking time between the eight groups. Furthermore, we compared the demography of males and females using the prediction of the sampling theory in a metapopulation (Wakeley and Lessard 61; De and Durrett 10; Stadler et al. 51). In particular, we investigated the shape of the gene genealogy within each population and in pooled samples to qualitatively estimate the product of effective population size and migration rate of the metapopulation (*Nm*). The shape of the genealogy does not depend on the neutral mutation rate (Wakeley 60), therefore it is possible to perform an unbiased comparison of the *Nm* values of any markers. Finally, we performed spatially explicit simulations to investigate the population structure at both mtDNA and NRY, modeling their molecular evolution properties as well as the environmental and temporal heterogeneity (such as the emergence of agriculture) of WNG.

In agreement with patrilocality and polygyny, we found a strong difference between male and female dynamics in WNG, both in terms of direction and rate of gene flow. The pattern of those differences and their magnitude emerged when analyzing data under a metapopulation approach, both indirectly by investigating the shape of the gene genealogy and directly by simulations taking environmental conditions and locus-specific characteristics into account.

## Material and Methods

### Population samples

**Figure 1:**
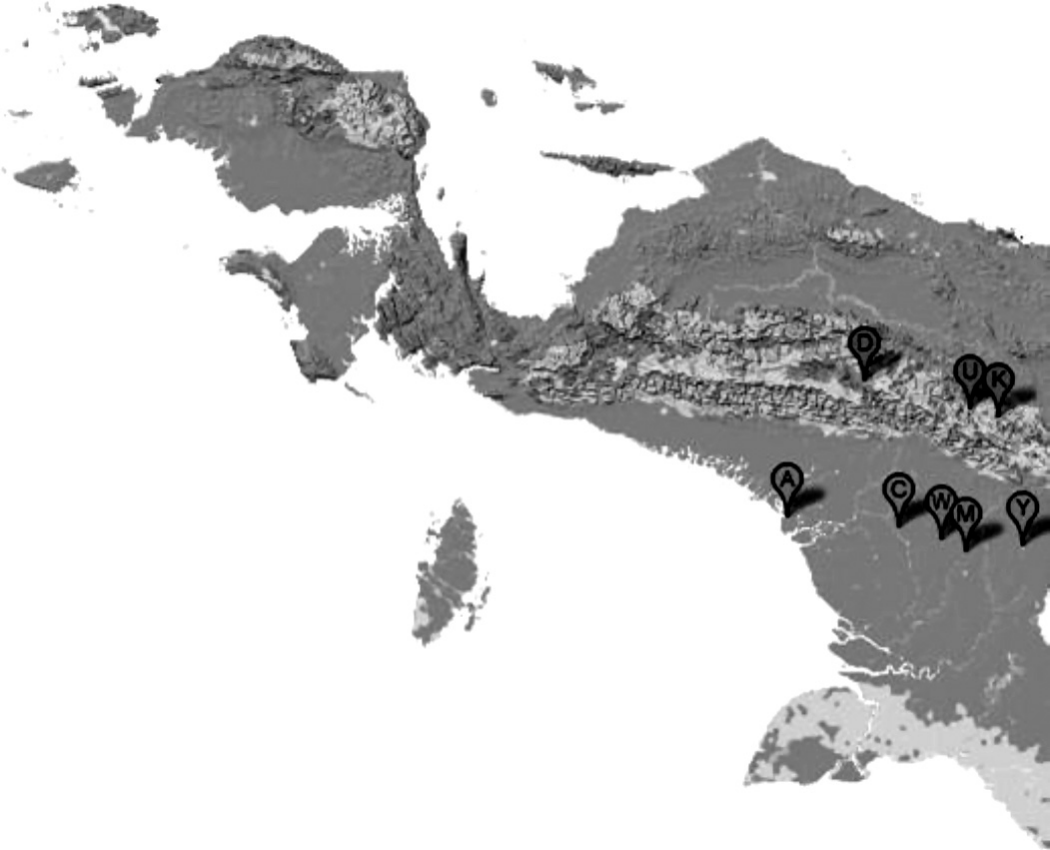
Geographic map of West New Guinea with the approximate location of the studied populations. *A*: Asmat; *C*: Citak; *D*: Dani; *K*: Ketengban; *M*: Mappi; *U*: Una; *W*: Awyu; *Y*: Muyu.

We included 200 mtDNA (Tommaseo-Ponzetta et al. 55) and 163 NRY (Kayser et al. 31) samples belonging to five populations of hunter/fisher gatherers (Asmat, Awyu, Citak, Mappi, Muyu) and three populations of agriculturalists (Dani, Ketengban, Una). The hunter/fisher gatherers groups (HG) inhabit the southern lowlands of WNG whereas agriculturalists (AG) inhabit the central and western highlands (Fig. 1). More details on these populations are available elsewhere (Tommaseo-Ponzetta et al. 55). We analyzed 350 bp of the hypervariable segment 1 of the mitochondrial control region (Tommaseo-Ponzetta et al. 55; data available from GenBank) and seven NRY microsatellites, namely DYS19, DYS389I, DYS389II, DYS390, DYS391, DYS392, and DYS393 (Kayser et al. 31, data available upon request). We did not use NRY haplogroup frequencies as haplogroup definition is based on a nonrandom sampling of single nucleotide polymorphism (i.e., resulting in ascertainment bias with unknown effect on the estimation of diversity and genetic distances).

### Isolation by distance

We computed two geographic distance matrices among the sampled populations. The first is the great circle distance, which represents the shortest line between two points and takes into account the curvature of the earth (GEO). The second is the distance in days of walk (DAY), which was computed by one of us (M. T.-P.) by traveling among all groups together with local guides. DAY implicitly reflects the environmental heterogeneities, as it represents the facility to move from one village to another. Pairwise genetic distances at mtDNA (*Fst*) and NRY (*Rst*) were computed using Arlequin 3.5 (Excoffier and Lischer 15). We refer to them hereafter as *Fst*_F_ and *Rst*_M_ to highlight that *Fst* was computed in females (from mtDNA) and *Rst* in males (from NRY). The correlations among the four matrices were estimated by pairwise Mantel tests, with the significance assessed after 10,000 permutations. Isolation by distance was further tested at both genetic markers by means of the Mantel correlogram (Legendre and Fortin 34) using GEO or DAY as a geographic distance matrix. The number of classes was determined using Surge's rule and significance within each class was assessed after 1000 permutations. All these analyses were performed using the libraries *ecodist* and *gdistance* implemented in the R statistical package (R Development Core Team 43).

### Indirect inferences on *Nm* values in a metapopulation

Human populations are almost never completely isolated but, rather, they belong to a metapopulation of demes exchanging migrants at various rates and with various dispersal kernels. The gene genealogy relating a sample of lineages extracted from a deme belonging to a metapopulation, as well as that of a pool of lineages taken from different demes, is mostly determined by the product of the effective population size *N* and the migration rate *m* (Wakeley 57, 58; Ray et al. 45; Stadler et al. 51). Contrasting the shape of the genealogy at various sampling level (i.e., single deme vs. a pool of lineages from different demes) provides indirect information on the absolute *Nm* value (Stadler et al. 51) independently of the knowledge of the mutation rate *μ*. The idea behind this approach is that in species experiencing range expansion (or more generally organized in a large array of subpopulations or demes) the coalescent history of a sample of lineages can be divided into a *scattering* and a *collecting* phase (Wakeley 56, 57). The relative length of the two phases determines the shape of the gene genealogy and it mostly depends on *Nm* (Wakeley 57; Ray et al. 45). This holds true for lineages sampled within a deme, scattered throughout the range of the species or pooled from various demes (Stadler et al. 51). Contrasting the shape of a gene genealogy in a metapopulation at all these sampling intensities can therefore provide information on *Nm*. High *Nm* values will determine signature of population growth at all sampling levels (Stadler et al. 51). For decreasing *Nm*, the signature of the range expansion will be lost first when sampling lineages from a single deme, then when pooling several lineages coming from different demes. The higher the number of lineages per deme pooled, the faster the signature of expansion will be lost. A scattered sample, where each lineage is extracted from a different deme, will be the last to lose such signature (Wakeley 57). Investigating the shape of the gene genealogy at various sampling levels is preferable to the comparison of fixation indices such as *Fst* (Excoffier et al. 16) and *Rst* (Slatkin 49), commonly used to estimate *Nm*. These fixation indices are dependent on *μ* and on the molecular evolutionary characteristic of the markers under examination (Hedrick 24). Moreover, they are based on simplifying assumptions which do not always hold in real settings (Whitlock and McCauley 62), while metapopulation and sampling theory is robust to various population genetic models (Wakeley 57, 59; Ray et al. 45; Wilkins 64; Stadler et al. 51).

To determine the shape of the genealogy, we used a Bayesian-based coalescent approach and compared different demographic models by means of Bayes Factors (BF). We used BEAST (Drummond et al. 14) to contrast a constant size model versus the extended Bayesian skyline plot (Heled and Drummond 26) for mtDNA, and Batwing (Wilson et al. 66) to contrast a constant size model versus a constant population starting an expansion T generations before present for NRY. Default priors were used in BEAST, whereas priors as in Mona et al. (39) were set in Batwing. We ran all datasets with both software for 100,000,000 iterations with a 10% burn-in and a thinning of 1000. Convergence was checked by running each dataset twice and by reaching an effective sample size higher than 200 for all parameters in each analysis. Marginal likelihood was evaluated using the harmonic mean estimator (Kass and Raftery 30). BF were computed as twice the difference of the natural logarithm of the marginal likelihoods and interpreted using the Jeffrey scale as reported in Kass and Raftery (30). The harmonic mean is a simple estimator of the marginal likelihood and some concerns have recently emerged on its performance (Baele et al. 1). The advantage of using the harmonic mean is its computational efficiency, which is important because we run a large number of model comparison analyses. The analyses were performed in: (i) each of the eight groups; (ii) the total pooled sample; (iii) 100 datasets of (a) 16 lineages obtained by resampling two lineages per village (group 2L); (b) 24 lineages obtained by resampling three lineages per village (group 3L); (c) 32 lineages obtained by resampling four lineages per village (group 4L). In total, we analyzed 309 datasets under two different models for the two markers, amounting to 1236 coalescent analyses. The reasoning behind resampling 100 times two, three, or four lineages per village was to obtain a BF distribution at each sampling intensity to assess the impact of stochastic variance in the coalescent process.

### Spatial explicit simulations

We performed a set of spatial explicit simulations of the demographic history of males and females in WNG using the software SPLATCHE (Currat et al. 9). SPLATCHE allows the simulation of a range expansion of haploid individuals over a two-dimensional array of demes arranged on a lattice and exchanging migrants with their four nearest neighbors. Simulations are done in two consecutive steps, namely the forward (demographic) and the backward (coalescent) steps. The forward simulation starts from an ancestral deme, which sends migrants to its neighboring demes. Migrations to empty demes represent new colonization events. Each deme has an intrinsic growth rate *g* and its density is logistically regulated by its carrying capacity (*N*) (Ray et al. 45). SPLATCHE uses a map of *N* values which can be also changed at user-defined time points. In this way, it is possible to model both spatial and temporal heterogeneity, by varying habitat quality (the *N* values) in space and time. After the regulation step, migrants are sent to the four neighboring demes at rate *m*. The process is repeated for successive generations for each nonempty deme, resulting in a wave of advance of the whole population. At equilibrium, each deme will send *Nm* migrants per generation to its surrounding demes. At each generation, the demographic and migration histories of every deme are stored in a database, which is then used in the backward coalescent step. The second phase of the algorithm then starts at the present generation, proceeding backward in time. The effective number of individuals present in a deme is used to compute the probability of a coalescent event, and the migration rates determine the probability of each sampled gene to emigrate, backwards in time, to the surrounding demes. The coalescent process stops after all genes have coalesced.

Spatial simulations were used to investigate under which demographic conditions we could reproduce the *Rst*_M_/*Fst*_F_ ratio observed in our data. Three of eight populations practice agriculture (AG groups), whereas the remaining five are hunter/fisher–gatherers (HG groups). Differences in *Nm* values between males (*Nm*_M_) and females (*Nm*_F_) can be due (among other factors) to different marital residence pattern and variance in reproductive success (e.g., due to polygyny). It is not known when these cultural behaviors arose in human populations and if they differ markedly between AG and HG groups (Marlowe 35). For this reason, we devised six evolutionary scenarios and tested a number of demographic parameters under each of them, considering also the possible increase in the carrying capacity of AG due to agriculture. The six scenarios are listed in Table 1: (i) *Scenario 1*: differences between *Nm*_M_ and *Nm*_F_ in HG, but not in AG. Postagriculture demographic expansion in AG; (ii) *Scenario 2*: differences between *Nm*_M_ and *Nm*_F_ in AG, but not in HG. Postagriculture demographic expansion in AG; (iii) *Scenario 3*: reduction in *Nm*_M_ compared with *Nm*_F_ in both AG and HG. Postagriculture demographic expansion in AG; (iv) *Scenario 4*: differences between *Nm*_M_ and *Nm*_F_ in both AG and HG. No postagriculture demographic expansion in AG; (v) *Scenario 5*: reduction in *Nm*_M_ compared with *Nm*_F_ in both AG and HG. These differences arose in all demes only after the emergence of agriculture. Postagriculture demographic expansion in AG; (vi) *Scenario 6*: reduction in *Nm*_M_ compared with *Nm*_F_ in HG. These differences arose in HG only after the emergence of agriculture. Postagriculture demographic expansion in AG.

**Table 1:** The evolutionary scenario tested

Model	*Nm* HG	*Nm* AG	Agriculture^1^	Description
1	*Nm*_M_ ≠ *Nm*_F_	*Nm*_M_ = *Nm*_F_	Yes	Sex differences in HG but not in AG. Increased carrying capacity in AG due to agriculture.
2	*Nm*_M_ = *Nm*_F_	*Nm*_M_ ≠ *Nm*_F_	Yes	Sex differences in AG but not in HG. Increased carrying capacity in AG due to agriculture.
3	*Nm*_M_ < *Nm*_F_	*Nm*_M_ < *Nm*_F_	Yes	Sex differences in both AG and HG. Increased carrying capacity in AG due to agriculture.
4	*Nm*_M_ ≠ *Nm*_F_	*Nm*_M_ ≠ *Nm*_F_	No	Sex differences in both AG and HG. No effect of agriculture
5	*Nm*_M_ < *Nm*_F_^2^	*Nm*_M_ < *Nm*_F_	Yes	Sex differences in both AG and HG. These differences appeared only after the beginning of agriculture. Increased carrying capacity in AG due to agriculture
6	*Nm*_M_ < *Nm*_F_^2^	*Nm*_M_ = *Nm*_F_	Yes	Sex differences in HG. These differences appeared only after the beginning of agriculture. Increased carrying capacity in AG due to agriculture

We assumed agriculture started 8000 years B.P. Before agriculture, there is no difference in *Nm* between AG and HG.

1 Agriculture increased (“Yes”) or not (“No”) the carrying capacity in AG, but not in HG.

2 Difference between *Nm*_M_ and *Nm*_F_ begins only after the emergence of agriculture.

We set the beginning of the range expansion into New Guinea at 2000 generations ago, which roughly corresponds to the first human presence in the interior highlands at 49,000 years b.p. (Summerhayes et al. 53) assuming a generation time of 25 years (Fenner 18). For simplicity and because of the lack of data specific to New Guinea, we assumed the same generation time for males and females even though Fenner (18) suggested the value of 31 and 25, respectively, for ancient populations. For all scenarios and all demographic parameters, we first modeled a rapid colonization of the whole of WNG in approximately 100 generations and changed the *Nm* thereafter. This choice was made for two reasons: first, to ensure the same colonization time for both sexes, and second, to cope with the earliest human remains in Australia. Human remains are as old as 46,000 years b.p. (Hudjashov et al. 28), implying that the wave of advance through New Guinea must have been fast. We assumed the origin of the range expansion in the Bird's Head region (North West of New Guinea) as it was proposed to be one of the possible arrival areas of modern humans (Birdsell 6). We also ran a set of simulations placing the origin of the expansion in the southern region of West New Guinea, obtaining similar results. To calibrate *N* values, we used density data available from modern and ancient hunter–gatherers group (Steele et al. 52; Bocquet-Appel and Demars 7; Binford 5). By fixing the carrying capacity of HG groups to 40 (Currat and Excoffier 8), we varied the total number of demes of WNG in order to obtain a density of 0.16, 0.34, and 1 per square kilometer. These densities refer to the population size before agriculture, when all demes had the same carrying capacity (no difference between HG and AG groups). The value of 1 has been used as an upper bound as it exceeds the density range proposed for hunter–gatherer groups (Binford 5). All results presented are based on a density of 0.16, but no significant differences were obtained in the other two cases. Briefly, a density of 0.16 corresponds to 448 demes and a total effective male or female size of 17,920 (leading to a total number of inhabitants of 17,920 × 4 = 71,680 following the computation of Currat and Excoffier 8). Altitudinal values were used to approximately define the number of AG demes (as agriculture is mostly practiced in the interior highlands), which we set to 90 for the 0.16 density map. The effect of agriculture (scenarios 1, 2, 3, 5, and 6) was simulated by suddenly increasing the carrying capacity of AG demes at 8000 years b.p. (Denham et al. 13; Denham 12). The migration rates *m* were set to obtain an *Nm*_M_ and *Nm*_F_ ranging from 0.6 to 20 for HG and from 10 to 150 for AG (in scenarios involving an effect of agriculture). Mutation rate for the hypervariable region of mtDNA was set according to Soares et al. (50), whereas for NRY we used an average value of 0.002 per generation per locus, according to the median posterior distribution obtained after the runs in Batwing. We simulated genetic data from eight demes with the sample size and coordinate position of our eight populations (therefore, three AG and five HG groups). For each parameter combination under each scenario we performed 1000 coalescent simulations. *Fst*_F_ and *Rst*_M_ were computed with Arlsumstat (Excoffier and Lischer 15) and the averages were visualized by means of a contour plot. The maps corresponding to the three densities used are available upon request as raster map which can be imported in R using the *adehabitat* library.

## Results

**Figure 2:**
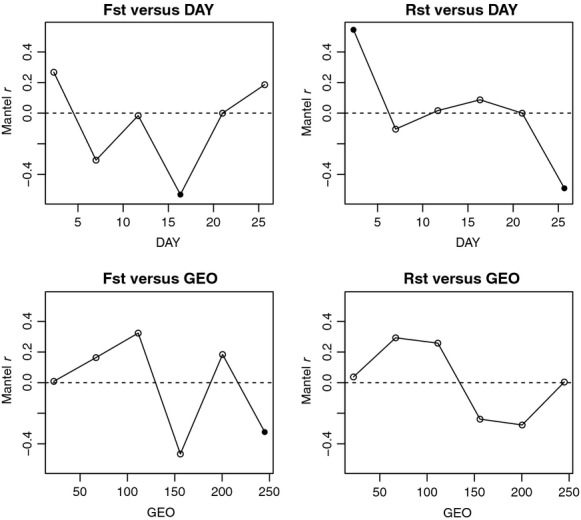
Mantel correlogram. *x-axis*: distance between groups in days of walking (DAY) or kilometers (GEO). *y-axis*: Mantel correlation coefficient. Black dots represent classes with significant correlation coefficient.

Despite being highly and significantly correlated (Table 2), DAY and GEO matrices behave rather differently when compared with both *Rst*_M_ and *Fst*_F_ pairwise distances. DAY is a good predictor of *Rst*_M_ matrix (0.693, *P* < 0.05), but not of *Fst*_F_ (Table 2). Moreover, the Mantel correlogram shows also a different pattern between the two markers, even though we note that the apparent clinal distribution of the NRY variation is mainly driven by the outlier Dani population (Fig. 2). Indeed, the correlation between DAY and *Rst*_M_ drop to 0.38 when excluding the Dani (*P* ≈ 0.20).Conversely, GEO is not correlated with any of the two genetic distance matrices (Table 2) and shows no clear trend when used in the Mantel correlogram analysis (Fig. 2).

**Table 2:** Pairwise Mantel correlation matrix

	GEO	DAY	Fst_F_	Rst_M_
GEO	–	*	n.s.	n.s.
DAY	0.521	–	n.s.	*
Fst_F_	0.193	−0.053	–	n.s.
Rst_M_	0.225	0.692	0.111	–

GEO, kilometric distance matrix; DAY, walking time distance matrix. Below diagonal: correlation coefficients. Above diagonal: significance of the test after 10,000 permutations. n.s., not significant.

* *P* < 0.05.

**Figure 3:**
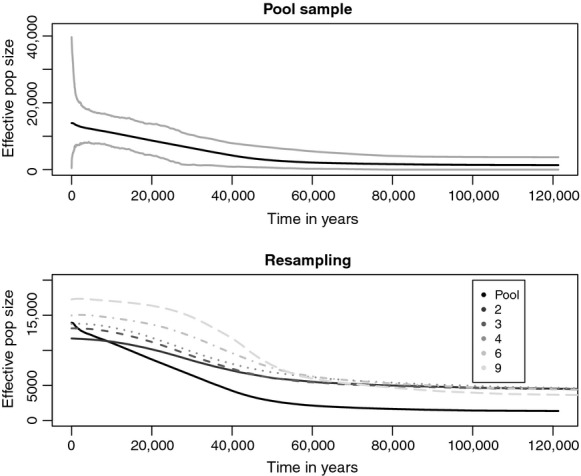
Extended Bayesian skyline plot computed in the: (A) pool sample; (B) Asmat; (C) Una. In (D), we plot the average skyline computed on 100 resampled datasets obtained by pooling 2, 3, 4, 6, or 9 lineages per population. Lines used are: solid, dashed, dotted, dot dash, long dash, or two dashes for the 2, 3, 4, 6, or 9 cases, respectively. *Ne* and coalescent times were scaled assuming the mutation rate reported in Soares et al. (50).

We computed the BF contrasting a constant size model versus the extended Bayesian skyline plot in the mtDNA dataset. None of the populations except Dani and Ketengban showed any support for one of the two models (Table 3). The pooled sample was no exception, with a BF of 0.49 in favor of the skyline model (Table 3). However, a qualitative comparison of the reconstructed variation in *Ne* through time highlights some differences between the single populations and the pooled sample (Figs. 3 and S1). Although each of the single populations displays a modest and recent signature of population expansion, the pooled sample experienced a strong demographic growth starting around 50 kya before present (assuming the mutation rate as in Soares et al. 50). This growth is well supported by the credible interval (Fig. 3) and coincides approximately with the arrival time of modern humans in New Guinea (Summerhayes et al. 53). We computed BF contrasting a constant size model versus an expansion model in the NRY dataset. According to the scale of Jeffrey, we found very strong evidence in favor of a constant population size model in all populations and particularly in the pooled sample (Table 3). We also reported the median and 95% credible interval of the *Ne* for all populations for both markers (Table 3). AG groups have lower NRY *Ne* compared with HG groups, while there is no trend in the mtDNA data (Table 3).

**Table 3:** Coalescent demographic estimate under a constant size model

Population	mtDNA					NRY								
	*n*	*k*	*Ne*	BF	*n*	*k*	*Ne*	BF
Asmat	25	17	4218 (1886–6921)	1.43	20	14	609 (160–1050)	71.23**
Awyu	14	11	6461 (2608–11,117)	0.72	10	6	282 (33–628)	27.11***
Citak	39	23	8475 (4610–12,240)	0.12	28	14	473 (130–796)	68.21***
Mappi	19	14	6628 (3214–11,063)	1.77	10	6	631 (114–1224)	51.61***
Muyu	9	8	4770 (1358–9655)	−1.70	6	5	697 (133–1375)	16.49***
Dani	21	17	6529 (3057–10,442)	6.24**	24	7	203 (45–387)	65.35***
Ketengban	23	10	3017 (1306–5098)	2.44*	19	7	165 (24–341)	41.73***
Una	50	28	7119 (4362–10,528)	0.04	46	11	182 (38–328)	90.10***
Pool	200	89	17,298 (10,566–21,242)	0.49	163	56	928 (468–1302)	1156.77***

*n*, sample size; *k*, number of haplotype; *Ne*, effective population size (in parentheses the 95% high posterior density); BF, Bayes factor. ^*^positive evidence; ^*^^*^strong evidence; ^*^^*^^*^very strong evidence in favor of one model according to Jeffrey scale. Positive values support the constant model, negative values the skyline. *Ne* values were obtained assuming the mutation rate reported in Soares et al. (50) for mtDNA and the priors used in Mona et al. (39) for NRY. Dani, Ketengban, and Una are AG and the other populations are HG.

We computed the BF in the resampled dataset and present the results in Table 4. At all sampling intensities, there are approximately the same number of dataset supporting a constant or Bayesian skyline model in mtDNA. Accordingly, the mean BF shows no correlation with the number of lineages sampled per population and in any case supports one of the demographic models tested (Table 4). We also plot the average skyline for all sampling intensities (Fig. 3). In accord with the pooled sample, all skylines support an expansion around 50 Kya, with a stronger signal for higher sampling intensities (Fig. 3). A completely different picture emerged for the NRY. In the 16 lineages case (group 2L) there are nine datasets supporting an expansion, but only one in the 32 lineages case (group 4L). All the resampled datasets support strongly the constant size model in the 48 lineages case (group 6L) (Table 4). Moreover, there is a positive correlation between the mean BF and the sampling intensities. This result indicates that by increasing the number of lineages per population, the shape of the gene genealogy resembles more what would be expected under constant demography. This means that there will be more coalescence during the scattering phase, which is what we expect for small *Nm* value. The difference in the shape of the gene genealogy between mtDNA and NRY is therefore consistent with an *Nm*_M_ much smaller than *Nm*_F_.

**Table 4:** Bayes Factor results on resampled datasets classified according to Jeffrey scale

Model	Support	mtDNA							Y-chr									
		2L^1^	3L	4L	6L	9L	2L	3L	4L
Constant	Very strong	0	0	0	0	0	0	0	18
	Strong	0	1	0	3	3	46	90	81
	Positive	26	27	36	37	40	36	9	1
Size change	Very strong	0	0	0	0	0	0	0	0
	Strong	0	0	0	3	0	0	1	0
	Positive	14	22	24	28	32	9	0	0
	None	60	50	40	29	25	9	0	0
Mean values		1.23	0.65	0.83	1.23	1.15	19.87	61.78	115.98

In the last row, we reported the mean BF over 100 resampled datasets.

1 Number of lineages sampled per population.

**Figure 4:**
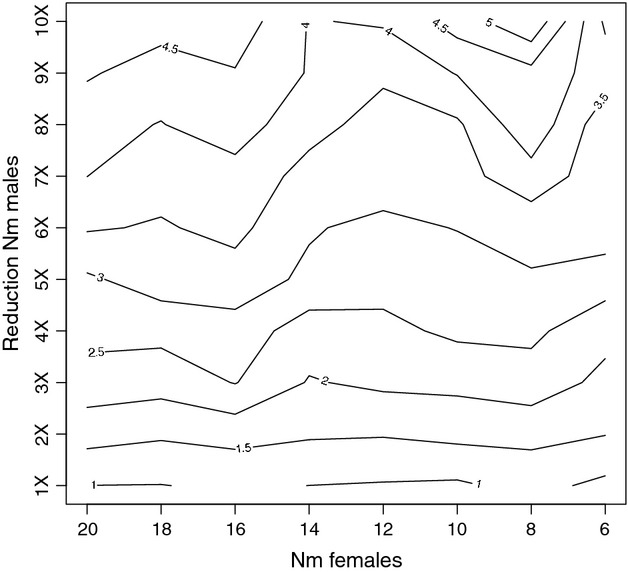
Contour plot obtained under Scenario 1 for *Nm* in AG = 150. *x-axis*:* Nm*_M_ of HG. *y-axis*:* Nm*_F_ of HG.

**Figure 5:**
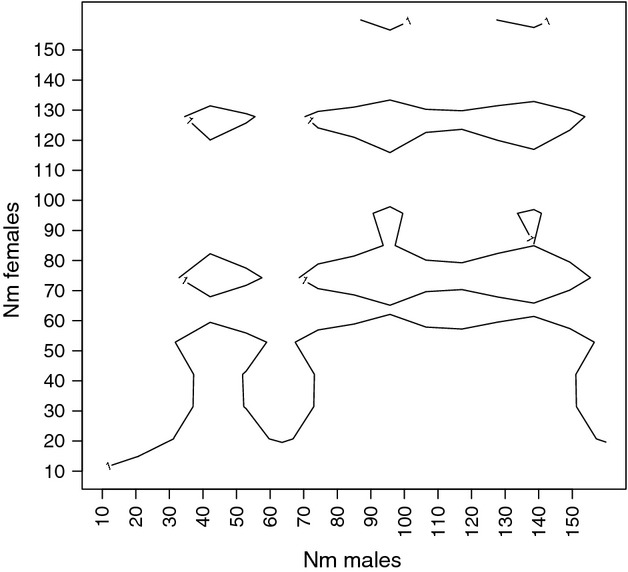
Contour plot obtained under Scenario 2 for *Nm* in HG = 1. *x-axis*:* Nm*_M_ of AG. *y-axis*:* Nm*_F_ of AG.

**Figure 6:**
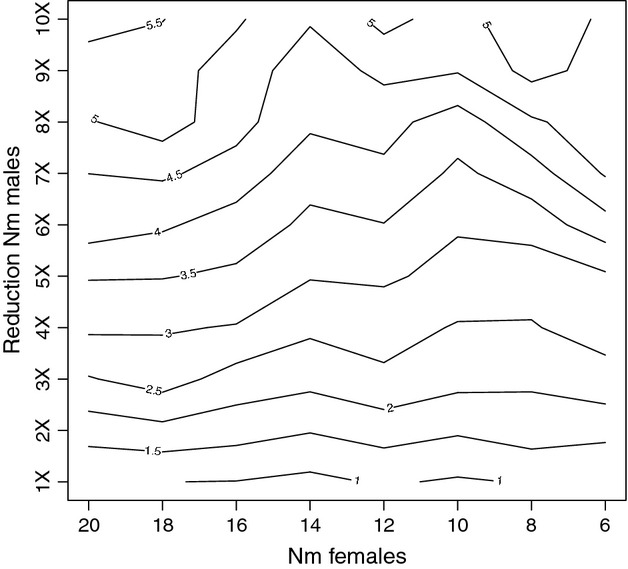
Contour plot obtained under Scenario 3 for *Nm* in AG = 150. *x-axis*:* Nm*_F_ of HG. *y-axis*: reduction intensity of *Nm*_M_ in HG and AG.

**Figure 7:**
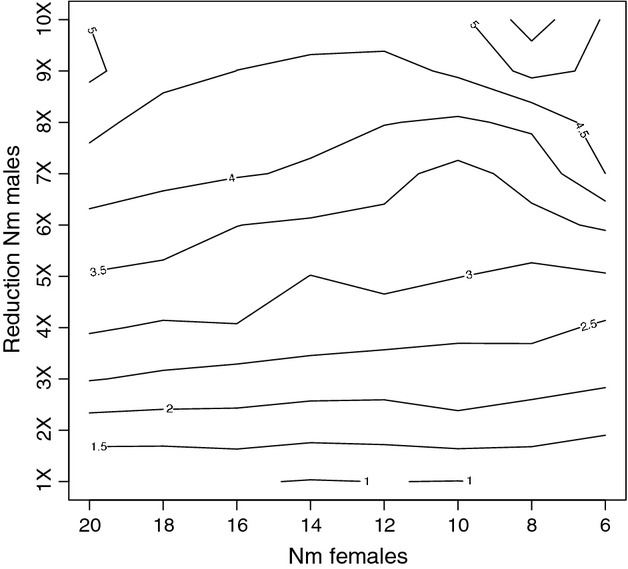
Contour plot obtained under Scenario 5 for *Nm* in AG = 150. *x-axis*:* Nm*_F_ of HG. *y-axis*: reduction intensity of *Nm*_M_ in HG and AG.

**Figure 8:**
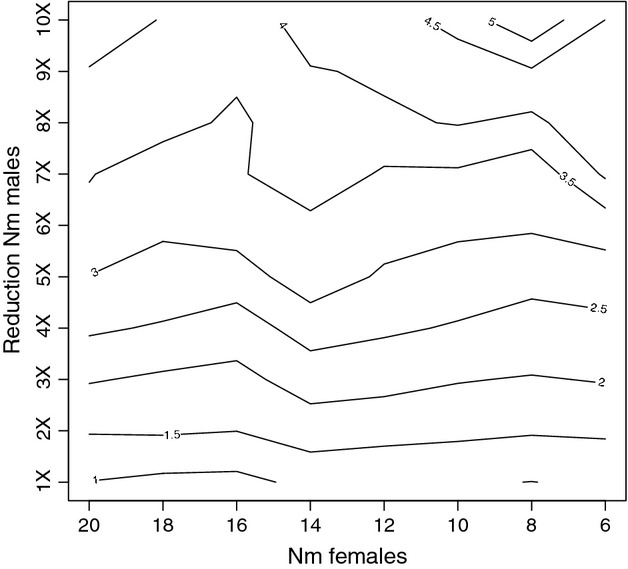
Contour plot obtained under Scenario 6 for *Nm* in AG = 150. *x-axis*:* Nm*_F_ of HG. *y-axis*: reduction intensity of *Nm*_M_ in HG and AG.

**Figure 9:**
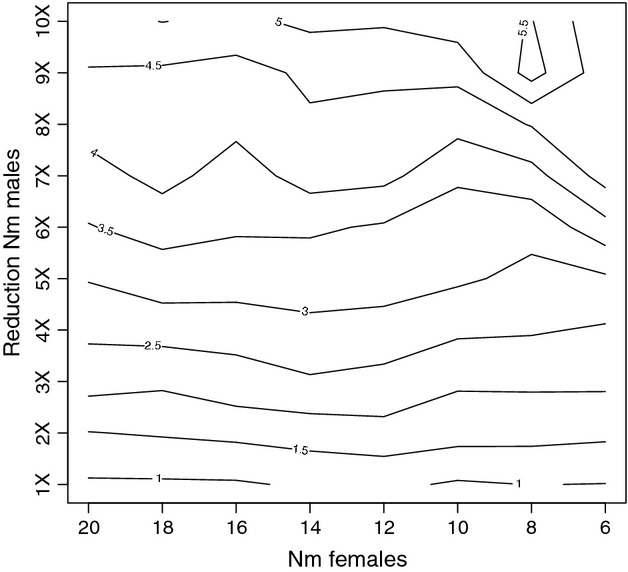
Contour plot obtained under Scenario 4. *x-axis*:* Nm*_M_ of AG. *y-axis*:* Nm*_F_ of AG.

We computed an *Rst*_M_ of 0.488 (*P* < 0.001) and an *Fst*_F_ of 0.116 (*P* < 0.001) among the sampled populations. The *Rst*_M_/*Fst*_F_ ratio is 4.2 which correspond approximately to an *Nm*_F_/*Nm*_M_ ratio of 7.26 assuming the Wright (68) island model. We tested more realistic and complex demographic scenarios by means of spatial explicit simulations. We aimed to determine under each evolutionary scenarios which values of *Nm*_F_ and *Nm*_M_ could reproduce an *Rst*_M_/*Fst*_F_ ratio around 4.2. Contour plots obtained under *Scenario 1* are presented in Figure 4. Under this hypothesis, we varied *Nm*_M_ and *Nm*_F_ of HG for fixed *Nm* in AG. We found that only a combination of *Nm*_M_ ≈ 1 (corresponding to an average *Rst*_M_ of 0.40 and 0.42 for *Nm* in AG of 150 and 20, respectively) and an *Nm*_F_ at least 10 times higher in HG is compatible with observed data, independently from the *Nm* in AG. Generally, reduction in *Nm*_M_ must be at least sevenfold larger when compared with *Nm*_F_ (Fig. 4 with *Nm* AG of 150; Fig. S2 with *Nm* AG of 20). Similarly, when we assumed that sex-specific differences occurred in HG only after the Neolithic we found still that an *Nm*_M_ reduction 10 times higher is needed to generate the observed *Rst*_M_/*Fst*_F_ ratio, independently from *Nm* in AG (*Scenario 6*, Figs. 8 and S6). In *Scenario 2,* we tested several combinations of *Nm*_M_ and *Nm*_F_ of AG for fixed *Nm* in HG. Under this hypothesis, we never found an *Rst*_M_/*Fst*_F_ ratio higher than 2 (Fig. 5, *Nm* HG of 1), independently on the *Nm* in HG (Fig. S3, *Nm* HG of 20). This result suggests that different behavior between males and females in West New Guinea are not (only) related to the emergence of agriculture as they must be present in HG as well in order to obtain such values of *Rst*_M_ and *Fst*_F_. *Scenario 3* and *5* assume that differences in the same magnitude between *Nm*_M_ and *Nm*_F_ are present both in HG and AG. In *Scenario 5,* such differences were established after the emergence of agriculture in all groups, whereas in *Scenario 3* they have been always present. In Figures 6 and 7, we plot the *Rst*_M_/*Fst*_F_ for various *Nm*_F_ in HG and an *Nm*_F_ = 150 in AG for scenario 3 and 5, respectively. In both scenarios, we found that a decrease in *Nm*_M_ of at least sixfold is needed to generate an *Rst*_M_/*Fst*_F_ ratio higher than 4, independently of the *Nm*_F_ set in AG (see Figs. S4 and S5 with *Nm*_F_ = 25 in AG). Finally, we presented in Fig. 9 the results of *Scenario 4* where we assumed no difference in carrying capacity between HG and AG (i.e., no effect of agriculture). We found a pattern quite similar to *Scenario 1* for various values of *Nm* in AG. However, we note that the absence of a Neolithic expansion increases the *Rst*_M_ and *Fst*_F_ values rather than their ratio, leading to an average *Rst*_M_ of around 0.71 for *Nm*_M_ = 1. This implies that an *Nm*_M_ ≈ 2 (leading to an average *Rst*_M_ of 0.40) and an *Nm*_F_ at least seven times higher is more compatible with our data.

## Discussion

Uniparental markers, namely mtDNA and NRY, have been used for a long time to characterize the influence of sex-specific behaviors on the genetic structure and diversity of human populations (Heyer et al. 27). Here, we addressed the general problem of comparing two loci with different mutational dynamics and we showed the benefits of accounting for the environmental features when investigating the spatial distribution of diversity. We used WNG as a case study for several reasons: (i) it is a region with complex environmental features and our samples come from heterogeneous areas; (ii) the eight populations under investigation are patrilocal. Polygyny is widespread too (Heider 25; Gardner and Weiner 21; Knauft 33), although the diffusion of these practices across the whole region is not homogenous and the importance of polygyny in generating sex-specific differences has been recently questioned (Heyer et al. 27). These characteristics are likely to generate sex-specific genetic patterns; (iii) reduced Y-chromosome but not mtDNA diversity in these populations has been already described (Kayser et al. 31), but no quantitative test was carried out to characterize male and female metapopulation dynamics.

We first note the importance of computing the real connectivity matrix relating the studied populations. The days of walking required to travel between groups are highly correlated with NRY pairwise distances (Table 2 and Fig. 2), even though it is important to highlight the role played by Dani, which are highly differentiated at NRY, but not at mtDNA and can drive such correlation (which drops to 0.38 and becomes not significant when removing them). At the same time, regardless of the measure of geographic distance used, there is no significant pattern in the distribution of mtDNA diversity. This finding would have not emerged by using kilometric distances only against which both markers exhibit a similar behavior (Table 2 and Fig. 2). In other words, by overlooking the true connectivity among populations we would have missed the differences in the migration trajectories between males and females. This finding is consistent with patrilocality, where males tend to live in the same village of their parents whereas females follow their husband (Marlowe 35). Males move following the least costly path according to the environment whereas females more randomly, probably as a consequence of being exchanged.

Not only the direction but also the intensity of gene flow differs between the two sexes. We found an *Rst*_M_/*Fst*_F_ ratio of about 4 which equals to a number of migrants per generation (*Nm*) about seven times higher in females than in males. This value is based on equilibrium model (Wright island model) which also overlooks the molecular properties of the two markers and their effect on the estimation of the fixation indices. To compare *Nm* between the chosen markers without worrying about their different molecular properties, we investigated the shape of the gene genealogy in all demes and in datasets created by resampling lineages from each of them. Range expansion is still visible in mtDNA, where resampled datasets support a growth around 50,000 years b.p. (Fig. 3), consistent with archaeological records (Summerhayes et al. 53). Contrary to what we expected from theory (Stadler et al. 51), we found a stronger expansion for higher sampling intensities. However, the power to detect an expansion using coalescent theory is correlated with the sample size for small datasets (Ramos-Onsins and Rozas 44). Therefore, the inferred skyline depends both on the true shape of the gene genealogy and the power to correctly recover it. The balance between these two factors also explains why there is no trend in the BF distribution. We found no signature of expansion in the single populations, which suggests that *Nm*_F_ must be lower than 20, as simulation studies showed expansions can be detected for *Nm* > 20 (Ray et al. 45). The picture for the NRY is drastically different, as only a few resampled datasets at the lowest intensity show a gene genealogy consistent with an expansion (Table 4). The signature of the range expansion into WNG has therefore been lost at the male level, which suggests a reduced *Nm*_M_. Even though, the resampling strategy we adopted does not provide a quantitative estimate of the *Nm*_F_/*Nm*_M_ ratio, it is an exploratory tool which has several important advantages over other approaches. Not only it can be used to compare different loci but also to get an accurate picture of the metapopulation dynamic. First, it does not rely on gene diversity or *Fst*, which means that any markers can be compared with and no need to know their evolutionary mechanism or rate. Here, we have analyzed two markers for which a wealth of information about their mutational processes is available from the literature, but we could easily adopt our approach to loci where knowledge is scanty even for humans. Second, it is robust to model misspecification, as it will hold as long as the coalescent process can be divided into a scattering and a collecting phase (which has been shown to work well for many metapopulation models [Wakeley 57; Ray et al. 45; Wilkins 64; De and Durrett 10]). This can free us from using *Fst* as an indirect measure of gene flow, as it has been shown that it works poorly under many conditions and even in equilibrium models (Whitlock and McCauley 62). Third, we got a time estimate of the range expansion occurred in New Guinea which we would have missed by analyzing each population separately only. Finally, we note that estimators of *Nm* values can be devised exploiting the resampling theory, for example by combining neutrality tests (compound tests) at various sampling intensities and/or using them as new and powerful summary statistics under an approximate bayesian computation inferential procedure. Coalescent approach coupled with sampling theory can be therefore applied to any structured species, provided that it is composed by a large (>100) number of demes.

To further investigate differences between *Nm*_M_ and *Nm*_F_ we also used spatial explicit simulations. The advantage of using simulations was threefold: first, we explicitly modeled the molecular dynamics of both mtDNA and NRY; second, we accounted for the genetic structure within WNG, including also the unsampled demes; third, we introduced in the population genetic model spatial and temporal heterogeneity (in particular, the effect of agriculture on the carrying capacity). For these reasons, we could not only make quantitative statements about the *Nm*_F_/*Nm*_M_ ratio, but also investigate when the differences arose. Independently of the scenario tested, we found that the observed *Rst*_M_/*Fst*_F_ ratio is compatible only with a number of male migrants per generation at least six times lower than female migrants (Figs. 9). Even though we could not infer precisely when this pattern of migration was established, there are some conclusions we could draw. Differences between male and female pattern must have emerged at least in the Neolithic, not only in AG but also in HG groups (Figs. 9). It is possible that, to generate the observed pattern, sex-specific behavior could have emerged even more recently than the Neolithic. However, we did not test for that as it would have increased the number of scenarios to analyze and there are no anthropological hypotheses supporting a more recent shift to patrilocality/polygyny. Many studies have suggested that HG groups are more flexible in terms of postmarital residence pattern, whereas AG groups are more predominantly patrilocal (Wilkins and Marlowe 65). Indeed, in our small dataset with only three AG and five HG populations we found an *Nm*_F_/*Nm*_M_ ratio of 14.6 and 1.5 for AG and HG, respectively, under the equilibrium island model. However, considering our more complex simulation framework, we could show that in WNG the differential behavior between males and females in HG is also necessary to generate the observed pattern of differentiation (see Figs. 5 and S3). Moreover, the *Rst*_M_/*Fst*_F_ ratio may have originated even assuming no sex-specific behavior in AG groups, provided that either the differences between males and females emerged early in HG groups (Fig. 4) or that they were particularly strong (i.e., *Nm*_F_ at least nine times higher than *Nm*_M_, Fig. 9). This result suggests that a key parameter to generate the difference between *Rst*_M_ and *Fst*_F_ is the number of demes having sex-specific migration pattern in a structured population: if only a small fraction of demes shift to patrilocality and/or polygyny, this will not significantly affect fixation indices. At the same time, if a large fraction of demes shift to patrilocality and/or polygyny, all demes of the metapopulation will be affected. To summarize, it is important to highlight the differences between estimating *Nm* through a spatial explicit model or an equilibrium model. Even if in our case results in terms of *Nm*_M_ and *Nm*_F_ values are very similar, this may have occurred by chance, as *Fst* is not an accurate estimator of *Nm* (see above). Moreover, we determined by simulations not only a quantitative difference in the number of migrants but also when social behavior started affecting the genetic variability in West New Guinea. Importantly, we also showed that many but not all populations need to have sex-specific behavior in order to affect the whole metapopulations. As a consequence, even populations with no sex-specific patterns will show differences between *Nm*_M_ and *Nm*_F_. Such a fine-scale characterization of metapopulation genetics could have not been possible using equilibrium based methods only.

Here, we have shown how to characterize sex-specific dynamics using both qualitative and quantitative approaches. Even though the specific pattern we observed could have been also influenced by more local events occurred at the single population level (i.e., the Dani and the Ketengban appears to be outlier at the NRY and mtDNA, respectively), the main message of this work is to show the importance of analyzing a structured species under a metapopulation framework and of introducing as much as possible the ecological features of an environment in the population genetics modeling. We did this by both performing spatial and temporal explicit simulations of genetic diversity and by applying indirect metapopulation inferential models. We showed how we can exploit prediction of the metapopulation theory simply by an appropriate sampling of genetic data, gaining a wealth of information (among others, the colonization time of New Guinea and the average connectivity among demes) which we would have missed under more traditional approaches. Moreover, the use of complex simulation schemes gave us the opportunity, not only of directly considering the connectivity among the sampled populations but also of introducing temporal and environmental heterogeneity and test their effects on genetic diversity. An extension to these analyses would be to implement approximate Bayesian computation (Beaumont et al. 3) to estimate separately the two parameters for both male and female, and to compare statistically the various demographic scenarios. Unfortunately, spatial explicit simulations are computationally intensive and more independent loci would be required for accurate parameter estimation (Bertorelle et al. 4). However, their flexibility should prompt them to become a useful tool to investigate a wide range of problem concerning structured populations.

## Data Accessibility

All the data used in this work have been previously published in Tommaseo-Ponzetta et al. (55) and Kayser et al. (31).

## Supporting Information

**Figure S1**. Extended Bayesian skyline plot computed in the each population. *Ne* and coalescent times were scaled assuming the mutation rate reported in Soares et al. (50).
