# Supplementary figures and images for: Investigating sex-specific dynamics using uniparental markers: West New Guinea as a case study
Source: Ecol Evol. 2013 Jul 2;3(8):2647–60. doi: 10.1002/ece3.660 (PMC3930047; doi:10.1002/ece3.660)

**Asmat**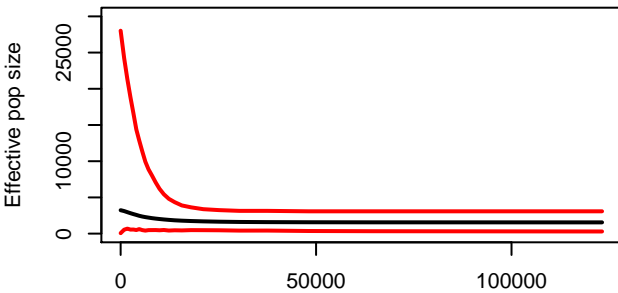**Awyu**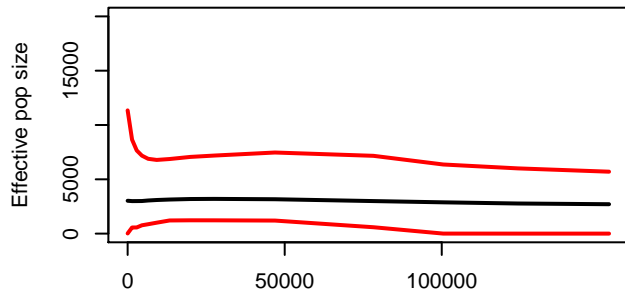**Citak**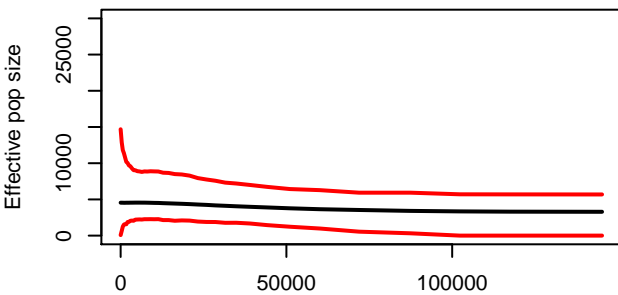**Dani**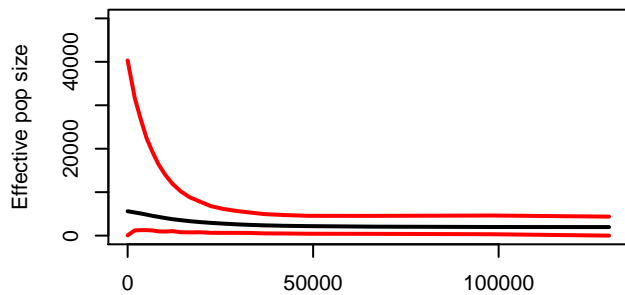**Ketengban**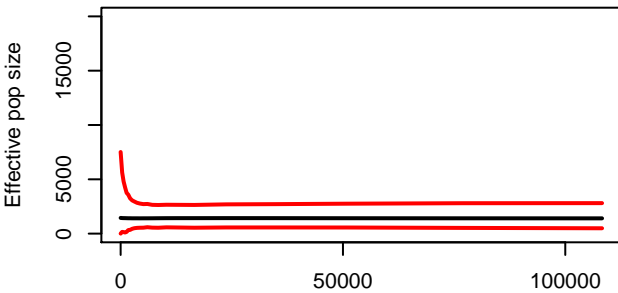**Mappi**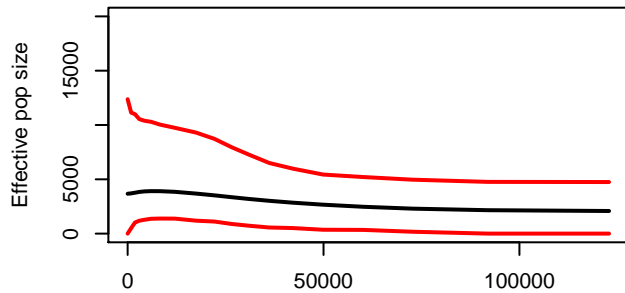**Muyu**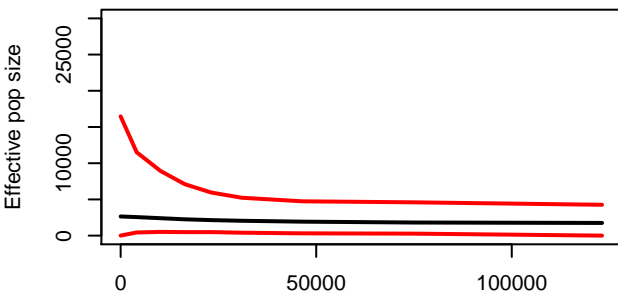**Una**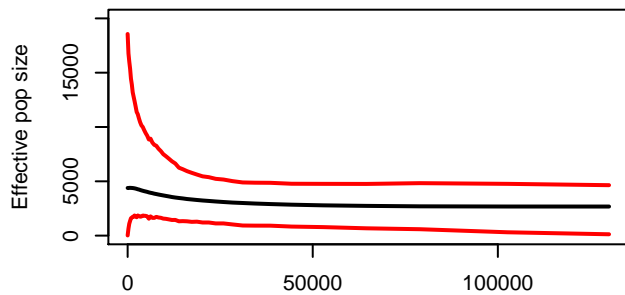

Supplement: Supplementary file 1 — Figure S1. Extended Bayesian skyline plot computed in the each population. Ne and coalescent times were scaled assuming the mutation rate reported in Soares et al. (50). [file ece30003-2647-SD1.pdf]

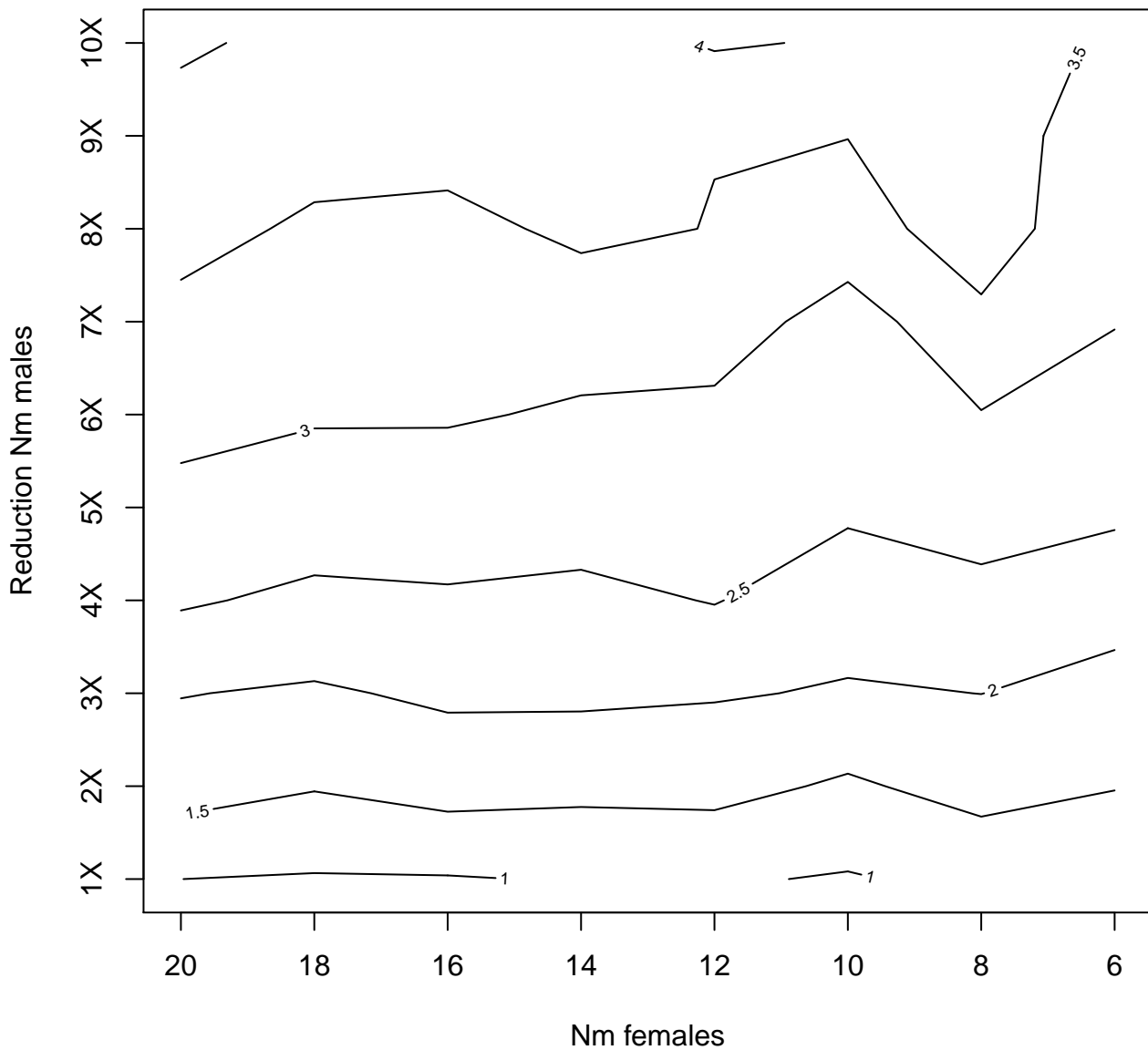

Supplement: Supplementary file 2 [file ece30003-2647-SD2.pdf]

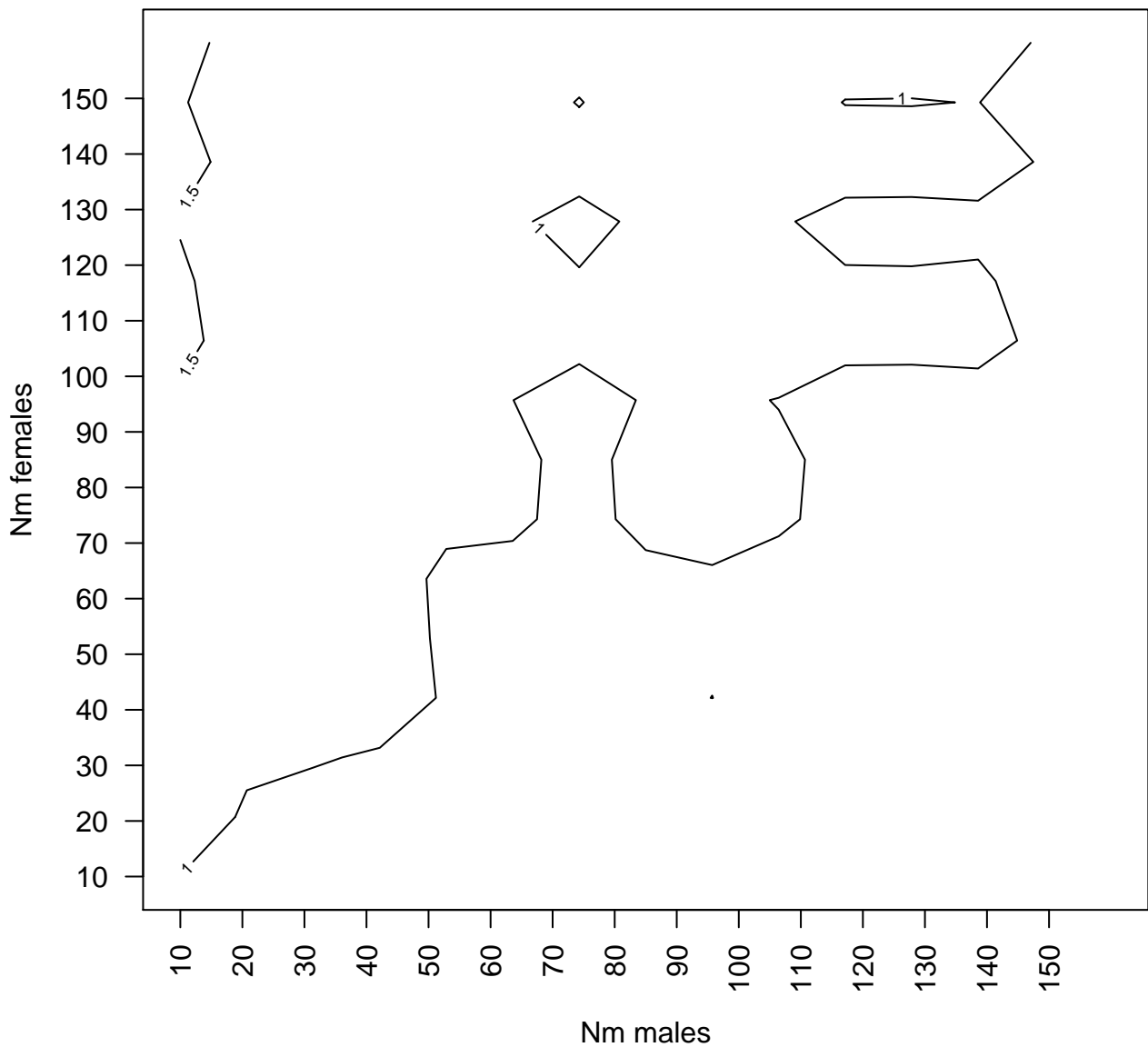

Supplement: Supplementary file 3 [file ece30003-2647-SD3.pdf]

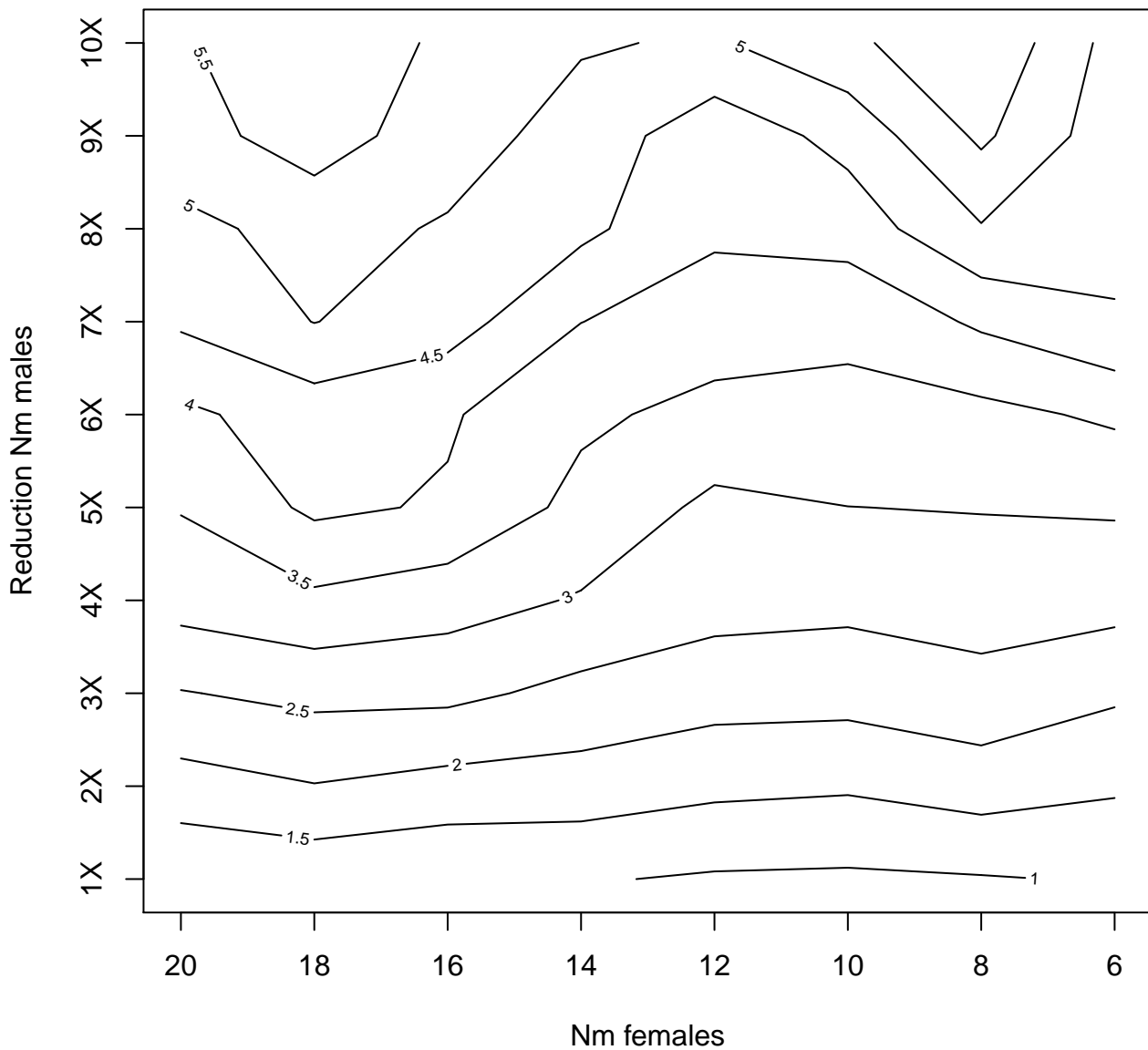

Supplement: Supplementary file 4 [file ece30003-2647-SD4.pdf]

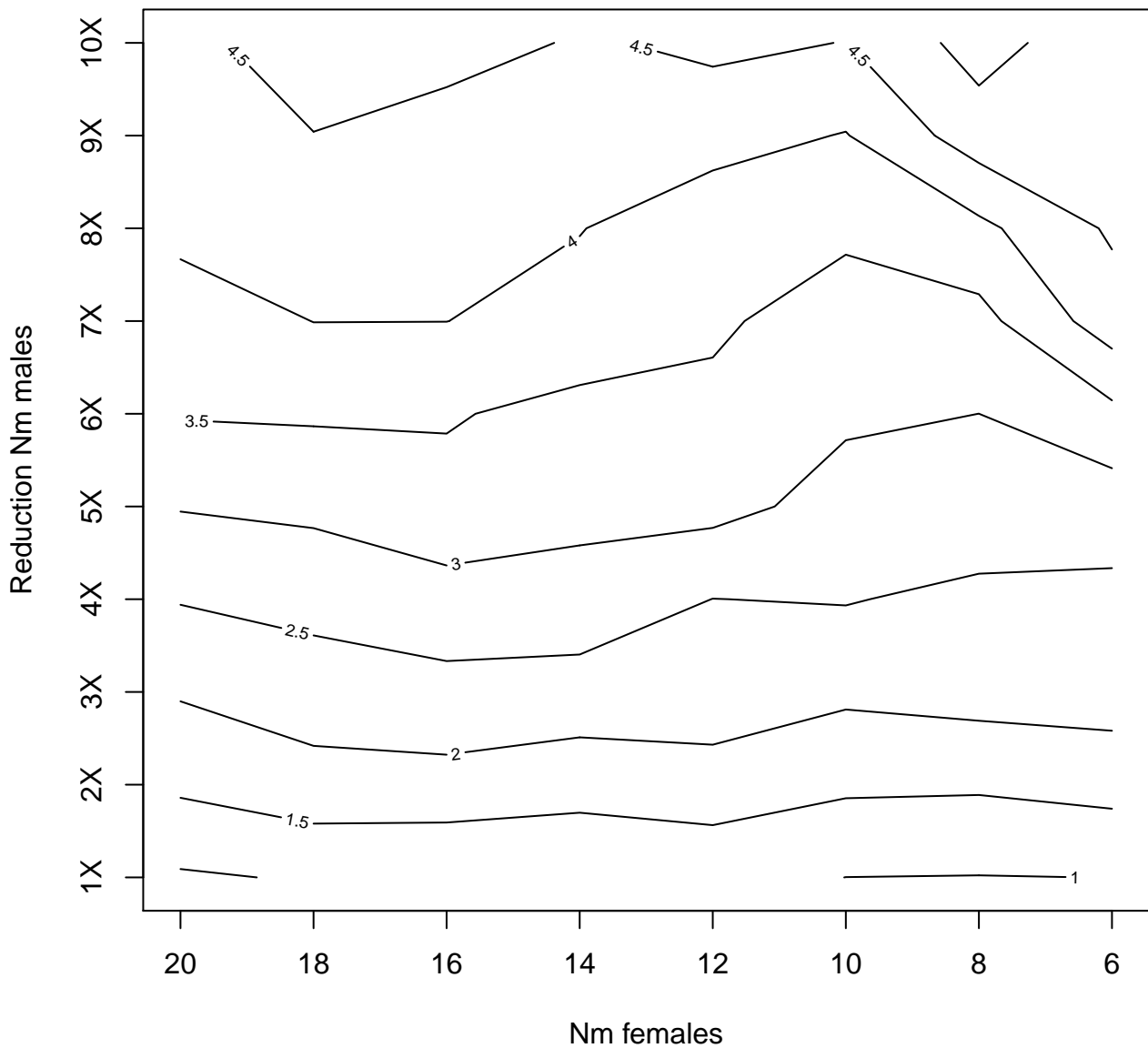

Supplement: Supplementary file 5 [file ece30003-2647-SD5.pdf]

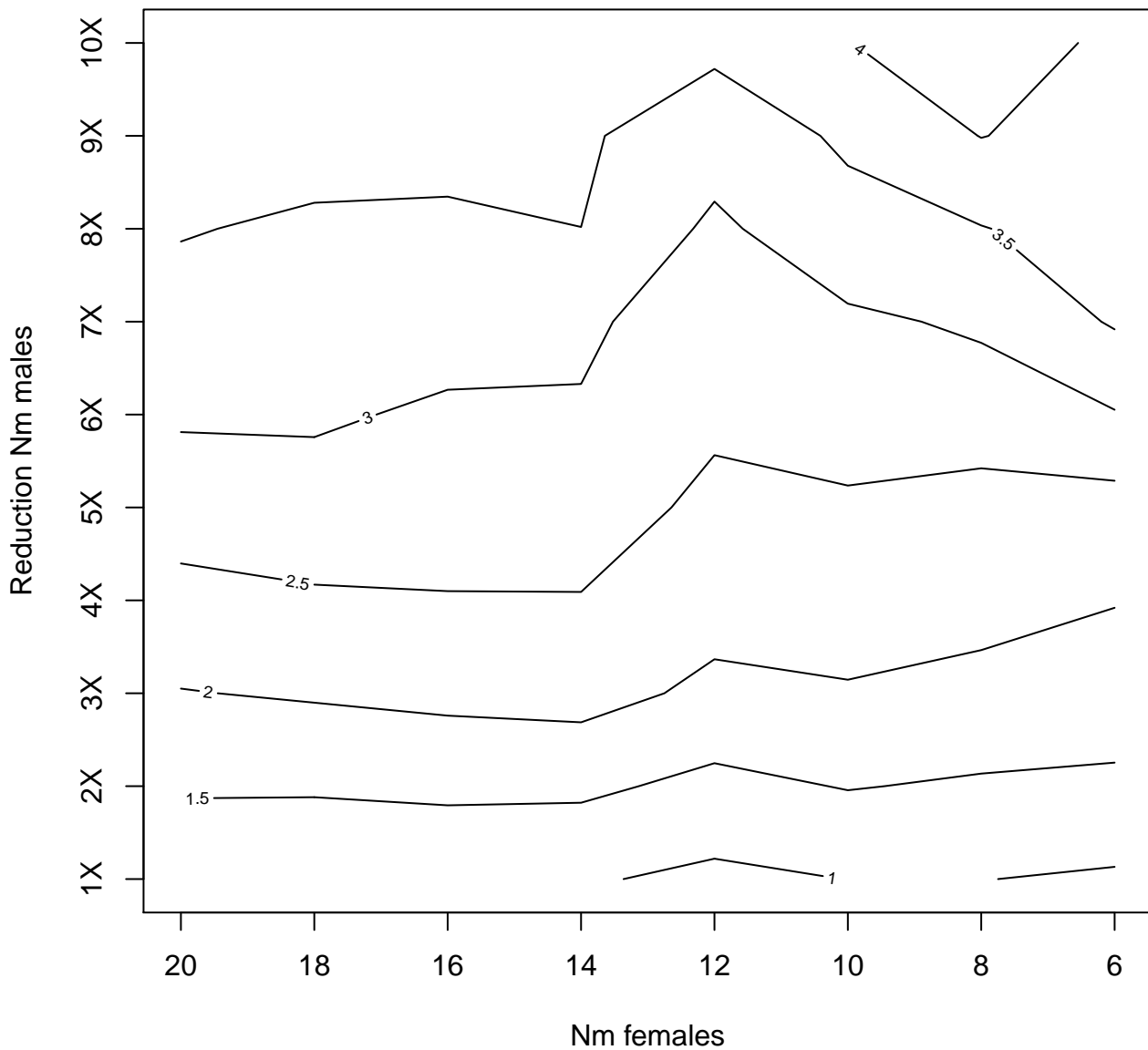

Supplement: Supplementary file 6 [file ece30003-2647-SD6.pdf]
